# Active learning for data-efficient segmentation of tea buds and leaves

**DOI:** 10.3389/fpls.2026.1865626

**Published:** 2026-09-16

**Authors:** Zhaodong Wang, Wenzhi Liao, Yang Li, Zhengyu Xiong, Faliang Wang, Kun Hu, Hao You, Qiang Xiao

**Affiliations:** 1 Research and Innovation Group of Detection Technology and Intelligent Control, School of Mechanical and Electronic Engineering, Jingdezhen University, Jingdezhen, China; 2 Department of Telecommunications and Information Processing, Ghent University, Ghent, Belgium; 3 Key Laboratory of Tea Quality and Safety Control, Ministry of Agriculture and Rural Affairs, Tea Research Institute, Chinese Academy of Agricultural Sciences, Hangzhou, China

**Keywords:** active learning, image segmentation, multi-view imaging, precision agriculture, tea bud detection

## Abstract

Accurate recognition and segmentation of tea buds and leaves is critical for precision tea management. However, developing robust vision models for tea bud and leaf segmentation typically requires large volumes of manually annotated data, which are costly and time-consuming to obtain. To address this challenge, we propose an active learning framework for data-efficient segmentation of tea buds and leaves. The proposed framework adopts an iterative training strategy that begins with a small set of labeled images and progressively improves the segmentation model by selectively annotating the most informative unlabeled samples. These samples are identified using mean confidence scores from a YOLO-based segmentation network, prioritizing uncertain instances to maximize learning efficiency. The proposed approach is evaluated using multi-view image datasets collected from tea plantations, including top-down and 45° perspectives, to investigate the influence of camera viewpoint on segmentation performance. In addition, segmentation accuracy is assessed across different tea plucking standards, including single buds, one-bud-one-leaf, and one-bud-two-leaves. Experiments show that, under the same annotation budget, the proposed framework achieves more than 10% higher accuracy compared to random sample selection. The performance gains are even more pronounced for 45° imaging, where our method outperforms random annotation by 14.7% in mAP@0.5 overall and by 19.0% specifically for bud detection.

## 1 Introduction

Tea is one of the most widely consumed beverages worldwide, with its quality and flavor strongly influenced by the selective harvesting of tender buds and young leaves. In premium tea production regions such as Jingdezhen, the plucking standard (single buds, one-budone-leaf, and one-bud-two-leaves) directly determines the final product’s sensory attributes and market value. Traditionally, tea harvesting depends heavily on skilled labor to identify and pick shoots at the precise growth stage. However, manual harvesting is increasingly constrained by labor shortages, rising costs, and inconsistency in human judgment Zhu et al. (2021). These challenges highlight the need for intelligent and automated harvesting systems capable of accurately recognizing tea plant components under natural field conditions.

Computer vision, particularly deep learning, has emerged as a powerful tool for agricultural automation, enabling high-precision detection, classification, and segmentation of plant organs. Early research on tea shoot identification primarily relied on handcrafted features based on color, texture, and shape. Wu et al. (2013) employed RGB chromatic differences between buds and mature leaves using an improved Otsu thresholding method for segmentation. Li et al. (2025) combined RGB-D information with spatial positioning to achieve three-dimensional localization of tea shoots. Jia et al. (2022) applied near-infrared spectroscopy and machine learning to classify Longjing tea quality through characteristic wavelength screening.

Recent progress in deep learning has substantially advanced automated tea shoot and bud recognition, with existing studies primarily spanning detection and segmentation paradigms. Region-based detectors such as Faster R-CNN have been used to classify tea shoot maturity stages, where Zhu et al. (2022) demonstrated that multi-label shoot recognition benefits significantly from enhanced backbone design. To improve real-time detection, several lightweight YOLO-based models have been proposed: Gui et al. (2023) introduced Ghost convolutions, BAM attention, and weighted feature fusion into YOLOv5 to achieve high accuracy with low computational cost, while Li et al. (2023) combined YOLOv5 with a Kalman filter tracker (ByteTrack) for robust video-based bud counting under occlusion. Beyond detection, segmentation networks offer precise localization for downstream tasks such as picking-point identification; for example, Hu et al. (2024) developed an improved DeepLabV3+ with MobileNetV2 and ECA-ASPP modules to enhance multi-scale feature fusion and robustness to illumination and occlusion.

Despite these advances, deploying deep learning models for tea harvesting in realworld plantations remains challenging. Tea plants exhibit complex canopy structures with overlapping leaves, fine-textured buds, and varying colors across developmental stages. In addition, environmental factors such as lighting, shadows, and wind-induced motion introduce further visual variability. The choice of imaging perspective also critically affects detection performance: a top-down view captures overall plant morphology but may obscure buds beneath leaves, while an oblique (e.g., 45°) angle provides richer three-dimensional cues at the cost of increased occlusion and background clutter. Determining the optimal viewing angle for reliable recognition thus remains an open question.

Moreover, developing accurate segmentation models typically requires large volumes of annotated data. For tea buds and leaves, manual annotation is time-consuming and labor-intensive, as fine-grained segmentation must distinguish subtle boundaries between overlapping structures. This data scarcity limits the scalability and adaptability of AI systems for different tea varieties and growth environments. Addressing this bottleneck demands more efficient annotation and training strategies that minimize labeling costs while preserving model performance. Recent advances in deep learning-based active learning (AL) have demonstrated substantial improvements in labeling efficiency and segmentation performance across a wide range of applications. In biomedical imaging, Zhao et al. (2025) proposed an AL pipeline that leverages both uncertainty- and diversitybased sampling to automatically identify the most informative samples with minimal human intervention, underscoring the effectiveness of hybrid selection strategies for complex instance segmentation tasks. Complementing this direction, Saran et al. (2023) introduced a streaming deep active learning framework in which diverse and uncertain gradient-based estimates guide sample prioritization, enabling faster model convergence and significantly reducing annotation requirements. In industrial settings, Liao et al. (2024) demonstrated that domain-tailored AL solutions can achieve high detection accuracy for gear defect analysis while dramatically lowering the need for extensive labeled datasets, highlighting the practicality of AL in real-world environments where annotation is costly and highly specialized.

Across these studies, state-of-the-art AL pipelines commonly integrate principled sampling strategies, based on uncertainty, diversity, gradient information, or their combinations, with robust modern segmentation architectures. Models such as Mask R-CNN He et al. (2017) and MaskFormer Cheng et al. (2021) are frequently employed to balance segmentation accuracy with computational efficiency. Empirical evidence consistently shows that uncertaintyaware metrics (e.g., entropy or confidence-based scoring), when combined with diversitypreserving selection, lead to more effective sample representativeness, faster learning curves, and reduced annotation demand. In many cases, competitive performance can be achieved using less than 30% of a fully annotated dataset, illustrating AL’s substantial potential for scalable and cost-efficient segmentation. AL has shown strong potential to reduce annotation costs in plant organ segmentation, as demonstrated by Rawat et al. (2022), where uncertaintybased sampling strategies significantly lowered labeling effort while maintaining competitive accuracy under different training configurations. However, their study focused on general datasets and did not address crop-specific harvesting standards, viewpoint variations, or the fine-grained distinctions required for tea bud segmentation.

The main contributions of this work are below:

1. Develop an active learning framework that reduces the annotation effort while achieving high-accuracy segmentation of tea buds and leaves.
2. To evaluate how imaging perspectives (top-down vs. 45°) influence recognition and segmentation performance.
3. Assess segmentation accuracy across different plucking standards (single bud, one-budone-leaf, and one-bud-two-leaves) and identify which are the most reliably detection by machine vision systems.

By systematically analyzing the impact of viewpoint and morphological complexity, this study provides insights into the feasibility and limitations of Active Learning-assisted model development for tea bud segmentation. The proposed framework demonstrates that AL can substantially reduce annotation costs while achieving competitive performance, establishing a methodological foundation for future work on annotation-efficient agricultural vision systems.

## 2 Materials and methodologies

Our study area is located within the high-standard tea plantation of Fu’an Tea Co., Ltd., situated in Fuliang County, Jingdezhen City, Jiangxi Province, China, as shown in **Figure 1**. The images were acquired on 8 April 2025 using an iPhone 15 Pro smartphone and were saved in JPG format with a uniform resolution of 1024×1024 pixels. The dataset comprises images of tender buds, one bud with one leaf, and one bud with two leaves, as shown in **Figure 2**.

**Figure 1:**
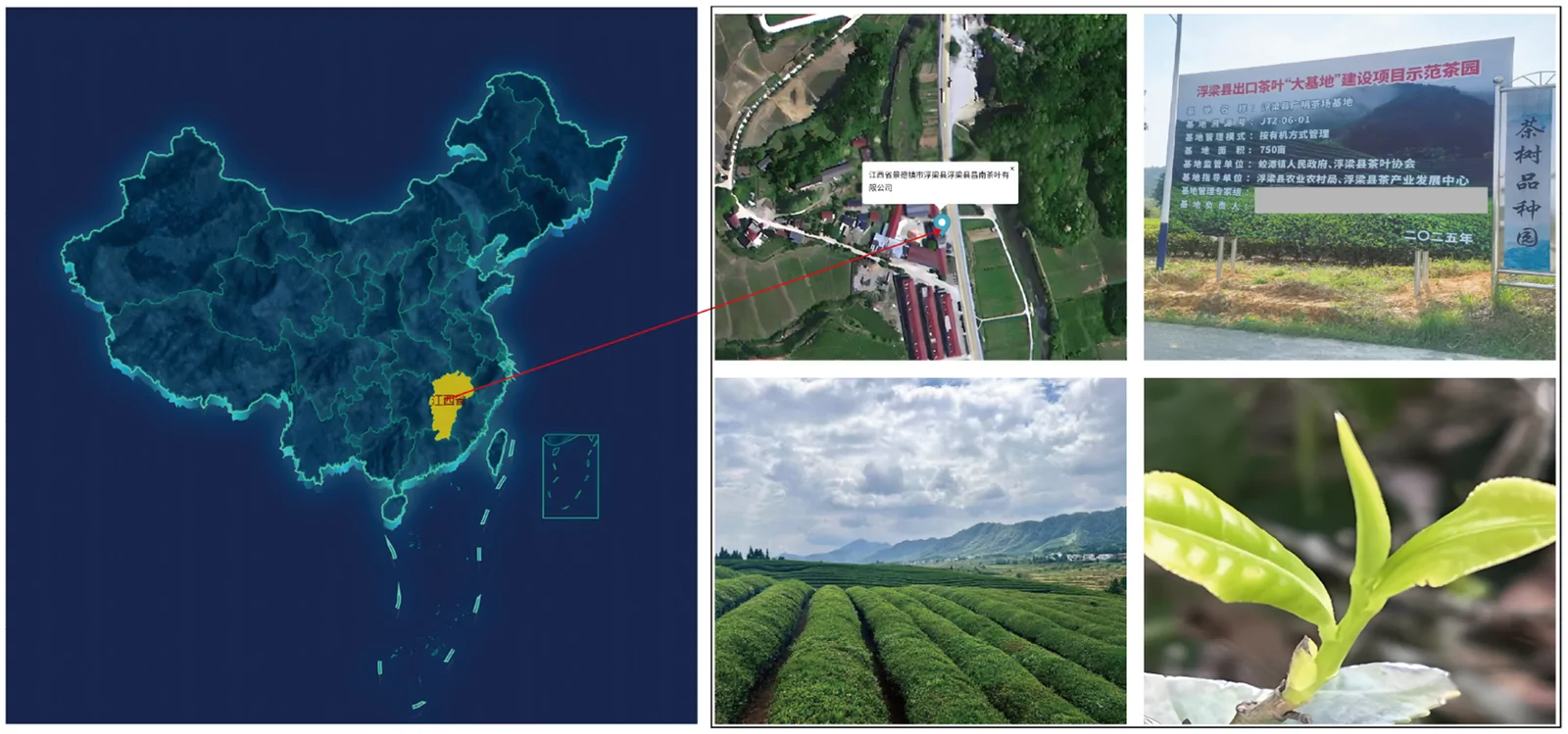
Study area for tea image data acquisition.

**Figure 2:**
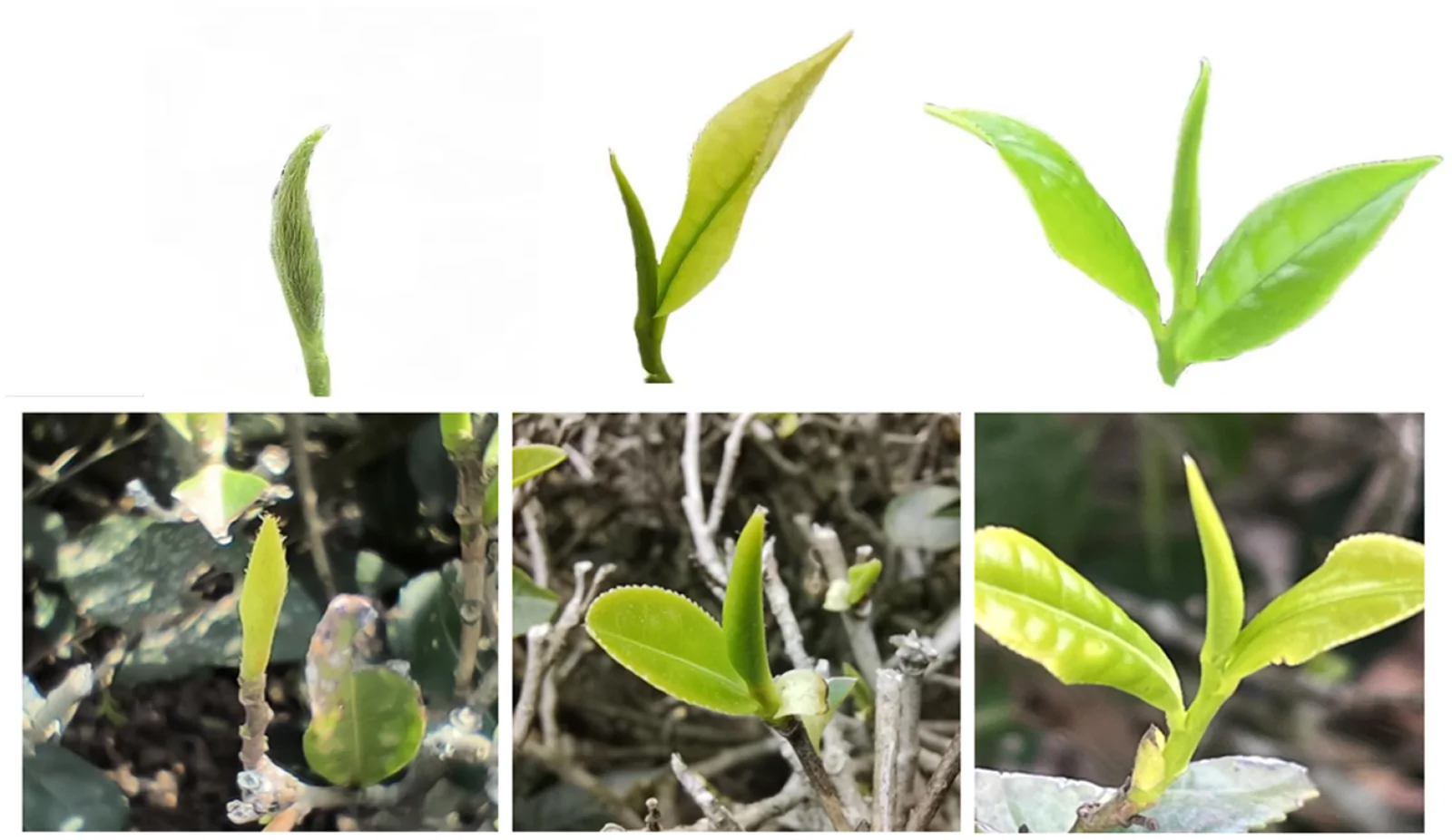
Three different bud, one-bud-one-leaf, and one-bud-two-leaves.

The production of Fuliang tea in Jingdezhen shows substantial differences in labor demand, cost structure, and economic return across plucking standards. Single-bud (“Bud”) harvesting is extremely labor-intensive, requiring nearly 20000 buds to obtain 500*g* of fresh leaves—and labor accounts for more than 60% of total production costs. In comparison, the one-bud– one-leaf (“Bud+1leaf”) and one-bud–two-leaves (“Bud+2leavs”) methods increase plucking efficiency by 3–5 fold, thereby reducing both labor input and overall production expenses. These disparities translate directly into market valuation: the price of “Bud” tea is roughly twice that of “Bud+1leaf” tea and about three times that of “Bud+2leaves” tea, reflecting the premium assigned to high-grade material and the diminishing value with increasing leaf inclusion.

The images were captured at either a 90° (vertical top-down view) or 45° (oblique view) angle, with shooting distances ranging from 10 cm to 60 cm, under natural daylight conditions to minimize shadow interference. A total of 8000 raw images were collected to construct the experimental dataset. These images encompass variations in background complexity, foreground distance (close-range vs. far-range), and target density per image. Close-range shots **Figures 3a, b** exhibit sparse targets with distinct foregrounds and low background complexity. In contrast, far-range images **Figures 3c, d** contain densely distributed and partially occluded buds, increasing detection difficulty and potentially leading to missed detections or false positives during segmentation and identification.

**Figure 3:**
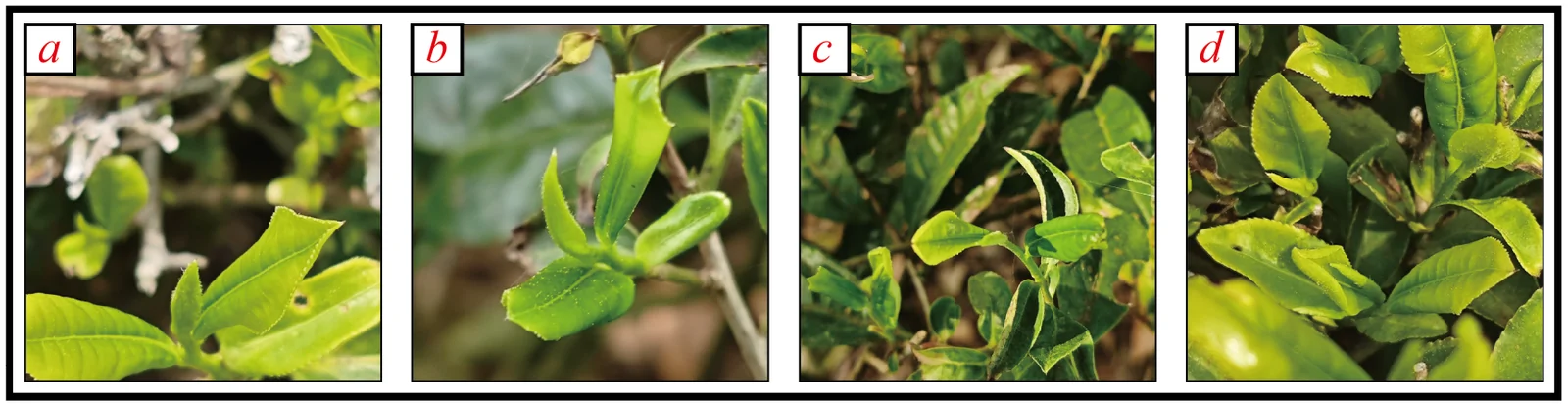
Examples of tea shoot images under different capture conditions: **(a, b)** closerange shots with sparse targets; **(c, d)** far-range shots with dense targets and higher background complexity.

The CVAT^^1^^ annotation tool was used to manually annotate 3 different categories: *Bud*, *Bud+1leaf*, and *Bud+2leaves*, aligning with actual harvesting standards.

### 2.1 Proposed method

Building upon these advances, this paper proposes an active learning-based segmentation framework for efficient labeling and segmentation of tea buds and leaves from field images. The objective is to minimize annotation cost while maintaining high segmentation performance by intelligently selecting the most informative samples for labeling. The framework integrates a lightweight segmentation model, YOLOv11 Khanam and Hussain (2024), with an uncertainty-based sampling strategy. YOLOv11 serves as the core segmentation backbone due to its strong balance of accuracy, inference speed, and computational efficiency, qualities essential for real-time agricultural applications. Unlike conventional encoder–decoder architectures such as DeepLab or U-Net, YOLOv11 adopts a single-stage design that jointly performs object detection and mask prediction. This enables precise instance-level localization of small and overlapping tea buds, while its nanoscale variant ensures suitability for deployment on edge devices such as mobile phones or field robots. Furthermore, the model’s integrated detection/segmentation structure facilitates efficient uncertainty estimation, which is critical for guiding iterative AL cycles. These characteristics make YOLOv11 an ideal foundation for the proposed framework, which performs iterative training, annotation, and inference under constrained labeling budgets and limited computational resources.

The proposed active learning framework follows the iterative process outlined in **Figure 4**. In particular, the overall workflow consists of four main components:

**Figure 4:**
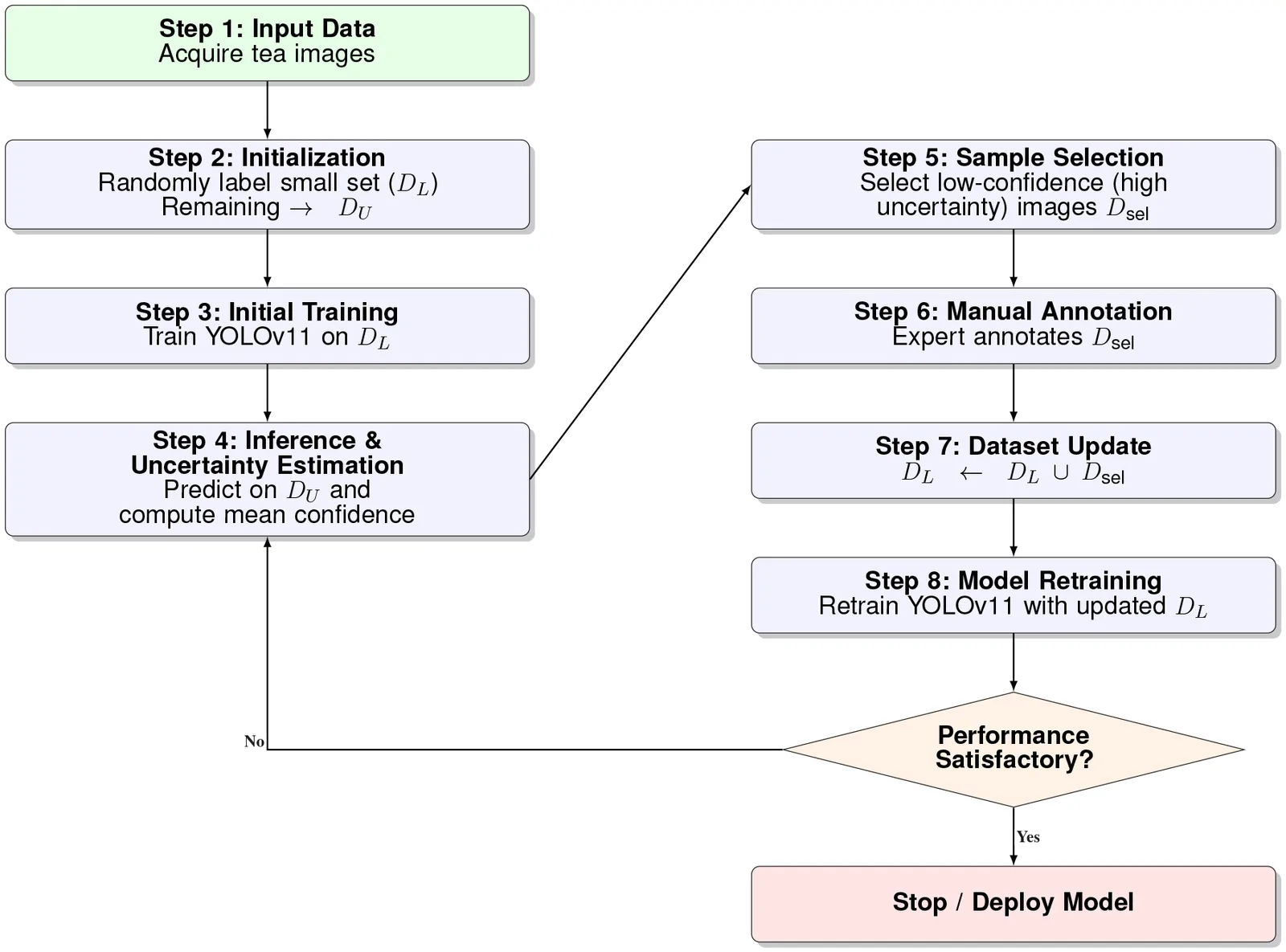
Flowchart of the proposed active learning-based tea bud and leaf segmentation framework.

1. Initial Model Training: A small subset of the dataset is randomly selected and manually annotated to create the initial labeled set. This is used to train a base YOLOv11 segmentation model.
2. Inference and Uncertainty Estimation: The trained model infers segmentation masks for the unlabeled image pool. For each image, class-wise confidence scores for detected tea buds and leaves are computed.
3. Sample Selection via Active Learning: Images are ranked based on their mean confidence scores. Low-confidence samples, representing high uncertainty, are prioritized for manual annotation.
4. Model Retraining and Iteration: Newly labeled samples are added to the training set, and the model is retrained iteratively until the segmentation accuracy is satisfied by the users.

This iterative cycle allows the model to generalize effectively with fewer labeled samples, forming a cost-efficient and scalable segmentation pipeline for tea bud and leaf analysis. The complete implementation is open-source and available ^^2^^.

#### 2.1.1 Proposed uncertainty-based sampling strategy

To identify the most informative samples for annotation in each iteration, we propose an uncertainty-based sampling criterion that quantifies the confidence of model predictions at the image level. The key idea is that images with lower mean confidence values indicate higher uncertainty and thus provide greater potential to improve the model when added to the labeled dataset.

For each unlabeled image $I_{i}\in D_{U}$, the trained YOLOv11 segmentation model outputs a set of *N_i_*detected instances $\left\{b_{1},b_{2},\ldots ,b_{N_{i}}\right\}$, each associated with a confidence score $c_{j}\in [0,1]$. The mean confidence score of the image is computed as Equation 1

(1) $$\bar{C}(I_{i})=\frac{1}{N_{i}}\sum_{j=1}^{N_{i}}c_{j}.$$

The proposed image-level uncertainty score is defined as the complement of the mean confidence, Equation 2:

(2) $$U(I_{i})=1-\bar{C}(I_{i}).$$

A higher value of *U*(*I_i_*) indicates greater prediction uncertainty, suggesting that the image contains more challenging samples, such as partially occluded buds, overlapping leaves, complex backgrounds, or low-contrast regions. During each active learning iteration, the *k* images with the highest uncertainty scores are selected from the unlabeled pool, Equation 3:

(3) $$D_{\text{sel}}=\underset{k}{\arg\text{ top}}\;U(I_{i}),\;I_{i}\in D_{U}.$$

These selected images are manually annotated and incorporated into the labeled training set *D_L_*for the next training iteration. By focusing annotation effort on the most uncertain samples, the proposed strategy improves annotation efficiency while reducing the overall labeling cost.

As a representative uncertainty-based baseline, we also evaluated an entropy-based active learning strategy. Instead of using only the predicted confidence, entropy considers the complete class probability distribution. For a detected instance with predicted class probabilities $p=(p_{1},\ldots ,p_{C})$ over *C* classes, the predictive entropy is computed as Equation 4:

(4) $$H=-\sum_{c=1}^{C}p_{c}\text{log }p_{c},$$

where larger entropy values indicate greater uncertainty. The image-level entropy score is obtained by averaging the entropy values of all detected instances as the Equation 5:

(5) $$\bar{H}(I_{i})=\frac{1}{N_{i}}\sum_{j=1}^{N_{i}}H_{j},$$

and the *k* images with the highest average entropy are selected for annotation. Unlike the proposed method, which relies only on the maximum prediction confidence, entropy exploits the full predictive probability distribution and therefore captures uncertainty arising from ambiguous class assignments.

The mean confidence metric was selected as the proposed acquisition function because it is computationally efficient and integrates naturally with the YOLOv11 detection and segmentation pipeline without additional probability calibration. Nevertheless, entropy, margin sampling, and variation ratio Settles (2009) capture complementary aspects of prediction uncertainty. Margin sampling evaluates the separation between the two most probable classes, while variation ratio measures prediction disagreement. Diversity-based methods, including core-set selection Sener and Savarese (2018) and BADGE Ash et al. (2020), further reduce sample redundancy by explicitly considering feature-space representativeness.

The proposed AL-based segmentation framework, integrated with the lightweight YOLOv11 segmentation model and the proposed uncertainty-based sampling strategy, offers several key advantages for efficient tea bud and leaf segmentation:

1. Reduced Annotation Cost and Improved Label Efficiency.

By prioritizing images with high prediction uncertainty for manual annotation, the proposed framework substantially reduces the number of samples required to achieve comparable or superior model performance. This selective labeling process minimizes redundant annotations of easily predictable images, leading to a more cost-effective data collection pipeline.

2. Enhanced Model Generalization Through Informative Sampling.

The uncertainty-based selection criterion ensures that the model is continuously exposed to challenging and diverse samples (e.g., occluded buds, complex backgrounds, or overlapping leaves). This iterative exposure prevents overfitting to easy cases and enhances the model’s robustness and generalization to unseen field conditions.

3. Lightweight and Scalable Implementation.

The integration of the efficient YOLOv11 segmentation model with the proposed AL strategy enables real-time inference and rapid retraining, making the approach suitable for large-scale field deployment. The framework’s modular design allows seamless adaptation to other crop types or segmentation tasks with minimal computational overhead.

### 2.2 Experimental design and statistical analysis

In this study, we designed a controlled experimental setup to evaluate the proposed active learning framework for tea bud and leaf segmentation under two camera viewing angles: a 45° oblique view and a top-down (90°) view, representing two common imaging perspectives in tea harvesting scenarios. **Table 1** summarizes the dataset composition for both viewpoints.

**Table 1:** Transparent breakdown of dataset composition per viewpoint.

Parameter	Oblique (45°)	Top-down (90°)
Initial annotated images	59	60
Fixed validation set	14	14
Initial training images	45	46
Unlabeled images (pool)	1200	1536
AL iterations	10	10
Images added per iteration	5	5
Total AL-added images	50	50
Final training set size	95	96

For the oblique (45°) viewpoint, 59 annotated images were collected, of which 14 were reserved as a fixed validation set and the remaining 45 images were used as the initial labeled training set. An additional pool of 1,200 unlabeled images was available for active learning. Similarly, for the top-down (90°) viewpoint, 60 annotated images were collected, including 14 validation images and 46 initially labeled training images, together with an unlabeled pool of 1,536 images. During active learning, the model was first trained using the initial labeled dataset. At each iteration, the proposed sample selection strategy identified the five most informative images from the unlabeled pool for manual annotation and inclusion in the training set. This process was repeated for ten iterations, resulting in 50 newly annotated images being added to the training dataset and yielding final training sets of 95 and 96 images for the oblique and top-down viewpoints, respectively.

All experiments were conducted using the official pretrained YOLO11n-seg.pt weights provided by the Ultralytics framework ^^3^^. The model was trained for 100 epochs per active learning iteration using a batch size of 4. To preserve consistency, we utilized the standard Ultralytics implementation default hyperparameter configurations; this includes standard inline data augmentations such as mosaic, HSV color space adjustments, and random horizontal flips. Experiments were performed on a Dell laptop workstation running Python 3.9.25 and PyTorch 2.8.0 with CUDA 12.8 acceleration, equipped with an NVIDIA RTX A500 Laptop GPU (approximately 4GB of available GPU memory) and 32GB of system RAM. The proposed active learning framework was implemented using the official Ultralytics training API. To ensure reproducibility, the complete implementation, including the active learning pipeline, training scripts, inference scripts, and all experimental settings, has been made publicly available at https://github.com/WenzhiLiao/active-learning-tea-segmentation.git.

The reasons that we selected the nano variant of Yolov11 are: (1) Active Learning requires repeated model retraining across multiple iterations, the nano variant offers substantially faster training times compared to larger variants, making iterative experimentation feasible within practical compute budgets. Given that the primary objective is to validate whether AL can accelerate annotation convergence rather than to achieve state-of-the-art accuracy, the nano variant provides a computationally efficient baseline for evaluating the relative improvement of AL over other methods; (2) The nano variant (approximately 2.6M parameters) is compatible with resource-constrained edge devices, ensuring that the methodological insights remain relevant to practical agricultural applications; (3) Lightweight YOLO variants have been widely adopted in agricultural detection tasks such as in Gui et al. (2023); Li et al. (2023), and our choice of nano variant of Yolov11 aligns with this established practice. Our Yolov11 nano segmentation model was trained using the default Ultralytics training configuration. Specifically, the optimizer was set to “auto”, with an initial learning rate (lr0) of 0.01. Unless otherwise stated, the remaining training hyperparameters were set to their default values provided by the Ultralytics framework.

In practical scenarios, users often select a limited number of images randomly for annotation due to time and labor constraints. To assess the effectiveness of our proposed active learning strategy, we compared it against a conventional random sampling approach, and entropy based method. In all cases, five new images were iteratively selected, annotated, and added to the training dataset in each iteration. This setup allows us to analyze how intelligently guided sample selection differs from purely random annotation in improving model performance and annotation efficiency over successive training iterations.

The annotated objects were categorized into three primary classes: “Bud”, “Bud + 1leaf”, “Bud + 2leaves”. The distribution of annotated objects across these classes is illustrated in **Figure 5**. From the figure, it can be observed that the “Bud + 2leaves” category dominates the dataset, accounting for over 50% of all annotations, while the other two classes contain substantially fewer instances. This class imbalance poses a challenge for model training and evaluation, as it may bias the segmentation model toward the majority class.

**Figure 5:**
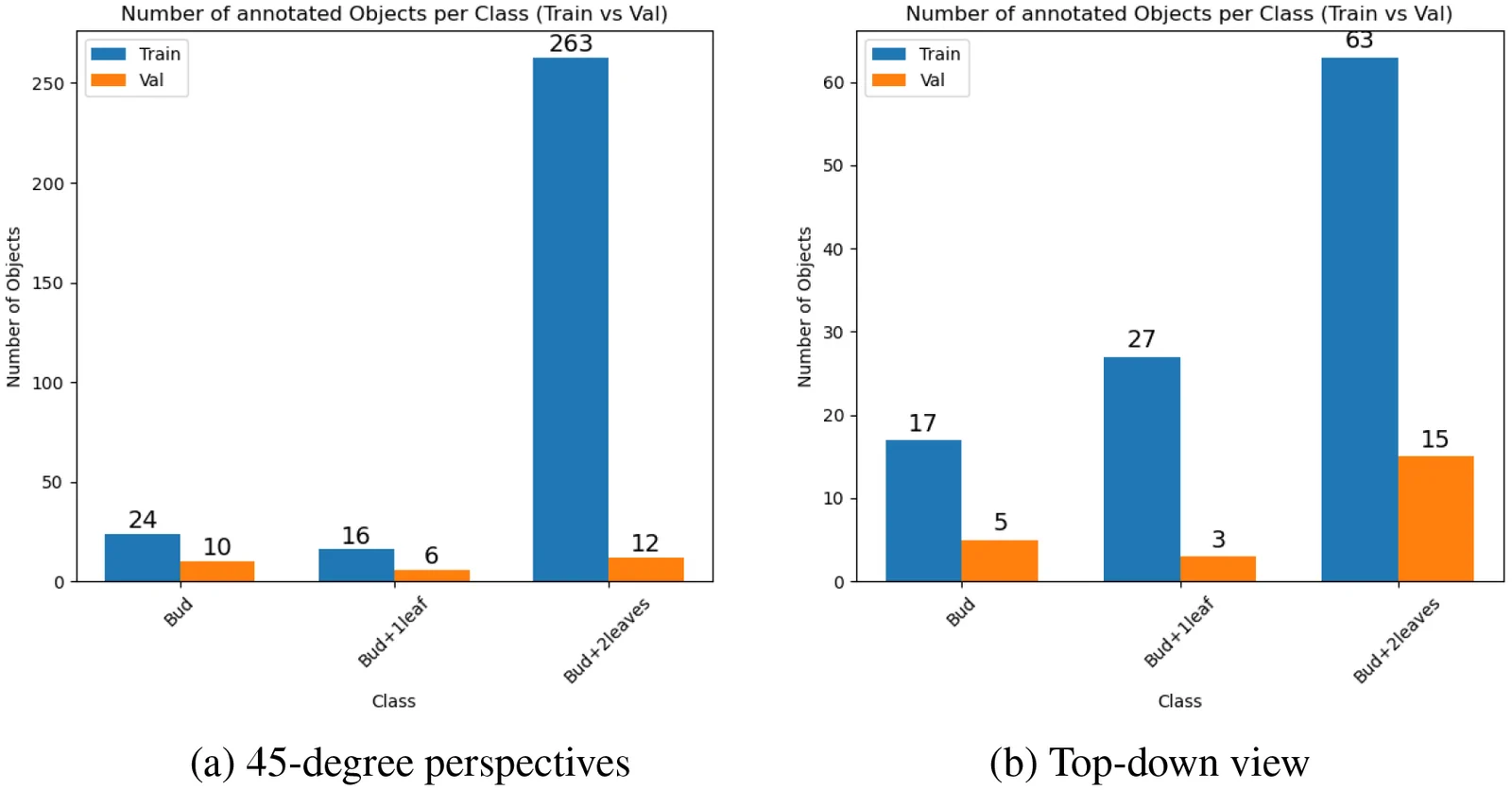
Number of annotated objects per class for images acquired by different angles. **(a)** 45-degree perspectives, **(b)** Top-down view.

To comprehensively assess the performance of the proposed active learning framework for tea bud and leaf segmentation, several standard segmentation metrics were employed, including mean precision (*P*), recall (*R*), and mean Average Precision (*mAP*). These metrics were evaluated both globally and per class to account for the class imbalance observed in the annotated dataset, where the “Bud + 2leaves” category constituted more than 50% of all samples (**Figure 5**).

*Precision* (Equation 6) quantifies the proportion of true positive detections among all positive predictions, reflecting the model’s ability to minimize false detections. *Recall* (Equation 7) measures the proportion of correctly identified objects relative to the total number of ground truth instances, representing the completeness of detection. *Mean Average Precision* (Equation 8) is computed as the mean of the average precision values across all target classes, providing an overall indication of detection and segmentation accuracy.

In addition, two widely adopted detection benchmarks were utilized: *mAP*@0.5, which calculates the mean average precision at an Intersection over Union (IoU) threshold of 0.5, representing detection accuracy under a permissive matching criterion; and *mAP*@0.5: 0.95, which averages the mean average precision over multiple IoU thresholds ranging from 0.5 to 0.95 in 0.05 increments, offering a stricter and more comprehensive evaluation of the model’s capability to accurately localize and segment tea buds and leaves under different viewing angles.

(6) $$P=\frac{1}{n}\sum_{i=1}^{n}\frac{TP_{i}}{TP_{i}+FP_{i}}$$

(7) $$R=\frac{1}{n}\sum_{i=1}^{n}\frac{TP_{i}}{TP_{i}+FN_{i}}$$

(8) $$mAP=\frac{1}{n}\sum_{i=1}^{n}\int_{0}^{1}P_{i}(R_{i})\;dR_{i}$$

Here, *TP* denotes true positives (correctly detected and segmented regions), *FP* represents false positives, and *FN* indicates false negatives.

## 3 Results

**Table 2** summarizes the segmentation performance of the YOLOv11 segmentation baseline model under two camera viewpoints: an oblique (45°) view and a top-down (90°) view. Overall, the top-down perspective consistently outperforms the oblique view across all evaluated metrics and object categories. Under the 90°configuration, the model achieves mean values of *P* = 0.484, *R* = 0.520, *mAP*@0.5 = 0.482, and *mAP*@0.5: 0.95 = 0.347, indicating a balanced trade-off between detection precision and recall. Among the three categories, Bud+2leaves achieves the highest precision (0.685) and mAP@0.5 (0.552), suggesting that larger, multi-leaf structures are more easily distinguishable from a vertical viewpoint. The Bud class also performs relatively well in recall (0.600), implying good detection coverage for simpler shoot structures.

**Table 2:** Segmentation performance of YOLOv11 baseline model under two viewpoints.

	Oblique (45°) view				Top-down (90°) view			
Metric	Bud	B+1L	B+2L	Mean	Bud	B+1L	B+2L	Mean
Precision (P)	0.311	0.289	0.572	0.391	0.447	0.321	0.685	0.484
Recall (R)	0.100	0.167	0.500	0.256	0.600	0.667	0.293	0.520
mAP@0.5	0.057	0.170	0.469	0.232	0.595	0.298	0.552	0.482
mAP@0.5:0.95	0.012	0.150	0.377	0.179	0.399	0.274	0.367	0.347

B + 1L = Bud + 1 leaf; B + 2L = Bud + 2 leaves.

In contrast, the oblique (45°) viewpoint yields lower segmentation performance, with mean values of *P* = 0.391, *R* = 0.256, *mAP*@0.5 = 0.232, and *mAP*@0.5: 0.95 = 0.179. The decline is most evident for the Bud category, where precision (0.311) and recall (0.100) are markedly low. This indicates that small or partially occluded buds are frequently missed under angled perspectives. The Bud+2leaves class performs slightly better (*mAP*@0.5 = 0.469), likely due to its larger and more distinguishable morphology. The overall degradation in accuracy highlights the challenges posed by perspective distortion, occlusion, and non-uniform illumination. These results emphasize that viewpoint geometry and dataset variability substantially affect segmentation outcomes. The limited number of annotated samples in the oblique dataset further constrains generalization, especially for small or infrequent object classes. Consequently, an active learning (AL) strategy is introduced to efficiently identify and annotate the most informative samples, thereby enhancing performance while reducing manual labeling effort.

In the proposed active learning framework, five unlabeled images were selected during each iteration according to the corresponding sampling strategy, manually annotated by an expert, and incorporated into the training set for the next learning cycle. **Figure 6** illustrates the segmentation performance throughout ten active learning iterations for the overall dataset and for each individual tea shoot category under both the oblique (45°) and top-down (90°) viewpoints. Across all experiments, the segmentation accuracy consistently improves as additional informative samples are incorporated into the training set, demonstrating the effectiveness of active learning in reducing annotation effort while progressively improving model performance.

**Figure 6:**
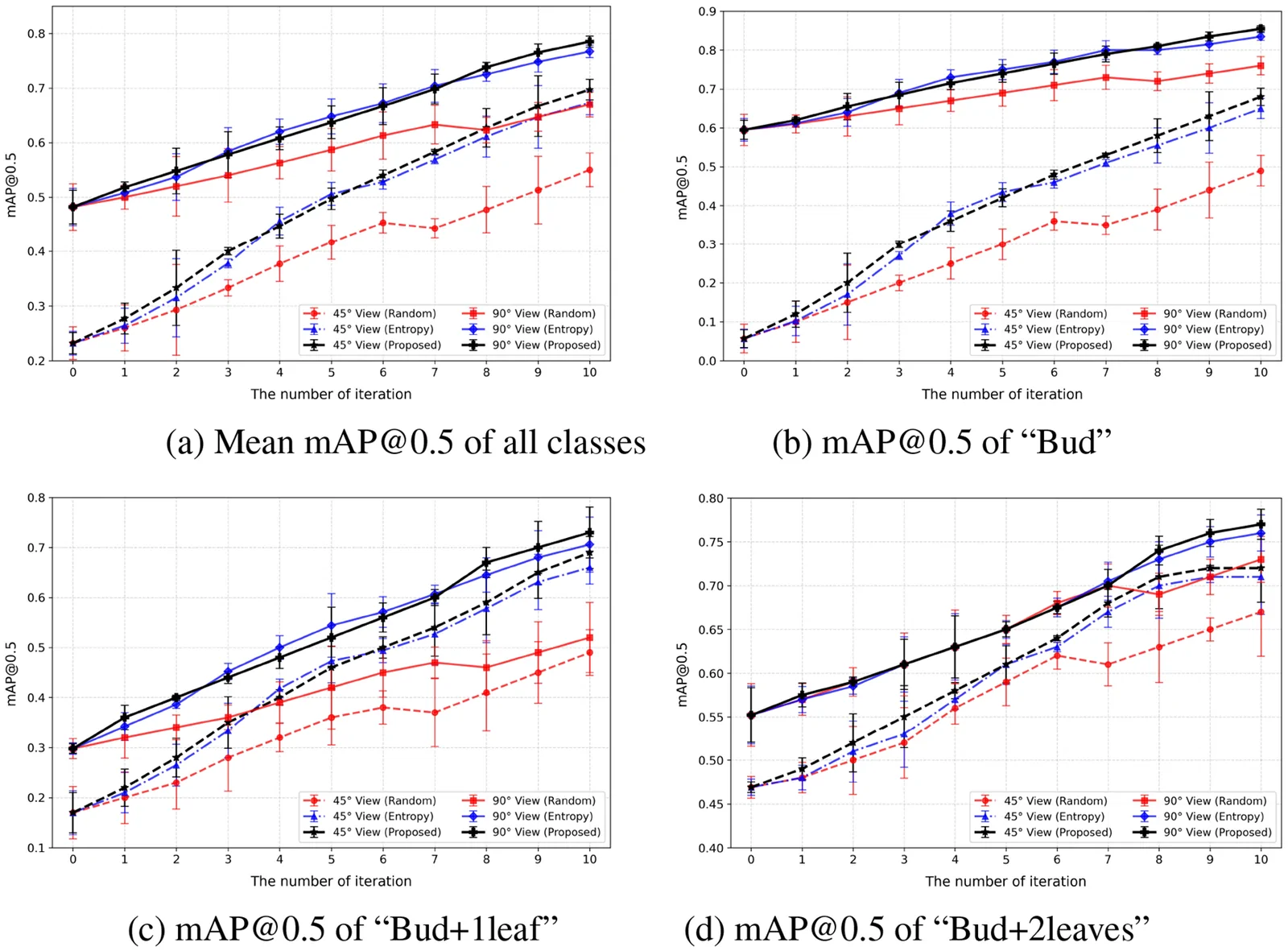
Average mAP@0.5 of the proposed method, entropy-based sampling, and random sampling over five independent runs. Error bars indicate ± one standard deviation. In each active learning iteration, five unlabeled images are selected for annotation and added to the training set. **(a)** Mean mAP@0.5 of all classes, **(b)** mAP@0.5 of “Bud”, (3) mAP@0.5 of “Bud+1leaf”, (4) mAP@0.5 of “Bud+2leaves”.

For a fair comparison, all methods were evaluated across five runs using different random seeds. **Figure 6** illustrates the mean mAP@0.5 values, with error bars representing ± one standard deviation across these repetitions. To evaluate computational efficiency, we measured the average image selection time per active learning iteration over a pool of more than 1500 unlabeled top-down tea images. Random sampling required only approximately 0.68 seconds per iteration, because no uncertainty estimation is performed. The proposed mean confidence-based strategy required an average of 7.91 seconds per iteration, as it computes only the average confidence scores of detected instances from a single forward pass of the YOLOv11 segmentation model. In comparison, the Entropy-based strategy required 12.76 second per iteration because it additionally computes class probability distributions and entropy values for every detected instance. The consumed selection time increase approximately proportionally with the size of the unlabeled sample pool, as more samples must be processed during each active learning iteration.

Compared with Random sampling, both uncertainty-based strategies exhibit faster convergence and consistently higher segmentation accuracy throughout the active learning process. For the overall segmentation performance (**Figure 6a**), the proposed method improves the mean mAP@0.5 from 0.482 to 0.785 for the top-down (90°) dataset and from 0.232 to 0.697 for the oblique (45°) dataset. The corresponding final mAP@0.5 values obtained by the Entropy-based strategy are highly comparable at 0.767 and 0.673, respectively, whereas Random sampling reaches only 0.670 and 0.550. The performance advantage of both uncertainty methods is particularly pronounced under the oblique viewpoint, where the increased scene complexity and occlusion make sample selection more challenging. In addition to achieving competitive final accuracy, the proposed method and the Entropy-based strategy converge with a similar rapid trajectory. Under the topdown viewpoint, the proposed method reaches approximately 0.70 mAP@0.5 by the sixth iteration, with the Entropy baseline matching this performance closely across iterations, while Random sampling fails to attain this accuracy within the evaluated budget. These results indicate that both uncertainty metrics successfully identify informative samples, enabling comparable model optimization profiles using the same number of annotated images. To provide a more tangible measure of annotation efficiency, we further estimated the number of randomly selected samples required to achieve performance comparable to that of the proposed active learning strategy. At the 5th active learning iteration, our method achieves a mean mAP@0.5:0.95 of 0.637, whereas Random sampling reaches a comparable performance only after approximately 8 annotation iterations. Assuming that the same number of images is added at each iteration, this corresponds to an estimated reduction of approximately 35% in the number of annotated samples. These results indicate that both the proposed and Entropy-based uncertainty strategies can identify informative samples and enable faster model optimization than Random sampling, achieving comparable or higher segmentation performance with substantially fewer annotated samples.

The class-wise evaluation presented in **Figures 6b–d** demonstrates similar observations. For the *Bud* category (**Figure 6b**), which represents the smallest and most challenging target, both uncertainty methods achieve strong, closely aligned trajectories under both viewpoints. Under the oblique (45°) view, the mAP@0.5 increases from 0.057 to 0.680 for the proposed method, compared with 0.650 for Entropy-based sampling and 0.490 for Random sampling. Under the top-down (90°) view, the proposed method reaches 0.855, while the Entropy and Random strategies achieve 0.835 and 0.760, respectively. These results demonstrate that uncertainty-guided sample selection, whether driven by mean confidence or entropy, is highly effective for improving the detection and segmentation of small or partially occluded tea buds.

A similar trend is observed for the *Bud+1leaf* category (**Figure 6c**). The proposed method achieves final mAP@0.5 values of 0.690 and 0.730 under the oblique and top-down viewpoints, respectively, compared with 0.660 and 0.706 for the Entropy-based strategy and 0.490 and 0.520 for Random sampling. While both uncertainty-based approaches perform comparably and substantially outperform Random sampling, the proposed method maintains a slight, steady advantage during the later active learning iterations, indicating that the selected samples remain highly informative as the labeled dataset gradually expands.

For the *Bud+2leaves* category (**Figure 6d**), all three sampling strategies achieve relatively high segmentation accuracy because the larger leaf structures provide more distinctive visual features. Nevertheless, both uncertainty strategies consistently deliver the best performance, with the proposed method reaching final mAP@0.5 values of 0.72 and 0.77 under the oblique and top-down viewpoints, respectively. The Entropy-based strategy follows closely with 0.71 and 0.76, whereas Random sampling reaches only 0.67 and 0.73. Although the absolute performance differences between the two uncertainty methods are minor, the proposed strategy consistently maintains a high-accuracy profile matching or slightly exceeding the entropy baseline throughout most active learning iterations.

The error bars in **Figure 6** demonstrate that both uncertainty-based sampling strategies exhibit similarly low variability compared to Random sampling across repeated experiments, indicating more stable and reproducible model performance. Overall, the proposed active learning framework and the entropy-based method consistently improve segmentation accuracy and data efficiency under both viewing angles. While achieving a closely matched segmentation performance with no significant difference from the Entropy-based strategy, the proposed method crucially reduces the average sample selection time by 60%, providing a vastly superior balance between computational efficiency, annotation effectiveness, and practical deployment. These results demonstrate that the proposed uncertainty metric is uniquely suited for scalable plant phenotyping applications, where minimizing runtime and annotation costs while maintaining peak segmentation performance is of primary importance.

**Figure 7** illustrates the first five images selected by the proposed active learning framework as the highest-priority samples for expert annotation. These images were chosen due to the high uncertainty exhibited by the initial model trained solely on the top-down view dataset. When applied to the new unlabeled test images, the initial model shows several consistent failure modes: large portions of the background are incorrectly segmented as Bud, and many Bud+2leaves structures are misclassified as Bud+1leaf. These errors reflect the model’s limited ability to generalize to unseen spatial arrangements and subtle morphological differences. Crucially, this selection behavior is heavily shaped by the dataset’s inherent class imbalance. Because the selection metric evaluates the mean confidence score across all detected instances within an image, samples dominated by the majority class (*Bud+2leaves*) yield higher overall confidence as the model rapidly masters these larger, visually prominent structures. Conversely, images containing rare classes (*Bud* and *Bud+1leaf*) are significantly more prone to lower mean confidence scores due to persistent model uncertainty, forcing the framework to preferentially prioritize them for manual annotation.

**Figure 7:**
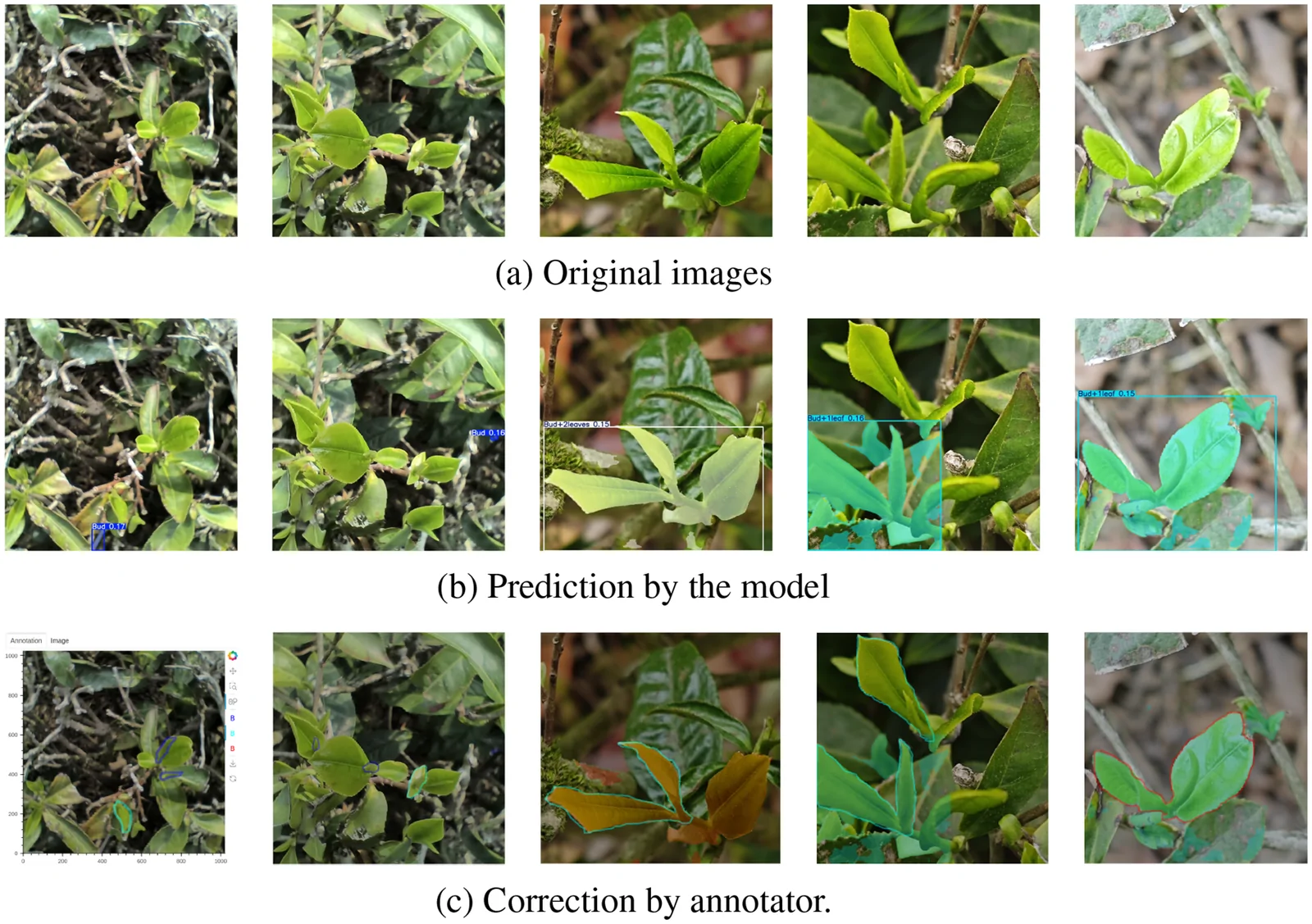
The first five images selected by the proposed active learning method **(a)**, predicted by the initial model **(b)**, and then interactively corrected by the annotator **(c)**. The correction takes around 2 minutes per image, the annotators interactively draw polygons, blue for “Bud”, cyan for “Bud+1leaf”, red for “Bud+2leaves”.

By manually correcting these segmentation errors, the newly annotated images are incorporated back into the training set, enabling the model to adjust its decision boundaries based on real and informative mispredictions. Each selected image requires approximately two minutes for refinement, resulting in a total annotation time of about ten minutes per active learning iteration, an efficient cost given the substantial accuracy gains observed in later stages.

The impact of integrating these five corrected samples is further demonstrated in **Figure 8**, which presents the normalized confusion matrices of the initial model and the model updated with the newly annotated images. Clear improvements can be seen in multiple categories, including reduced confusion between buds and background, and better separation between the Bud+1leaf and Bud+2leaves classes. These early improvements highlight the effectiveness of our uncertainty-driven selection, which creates a beneficial feedback loop: the framework naturally up-samples minority-class images, providing a self-correcting mechanism that mitigates class imbalance and guides the model toward the most informative training data without requiring explicit algorithmic modifications.

**Figure 8:**
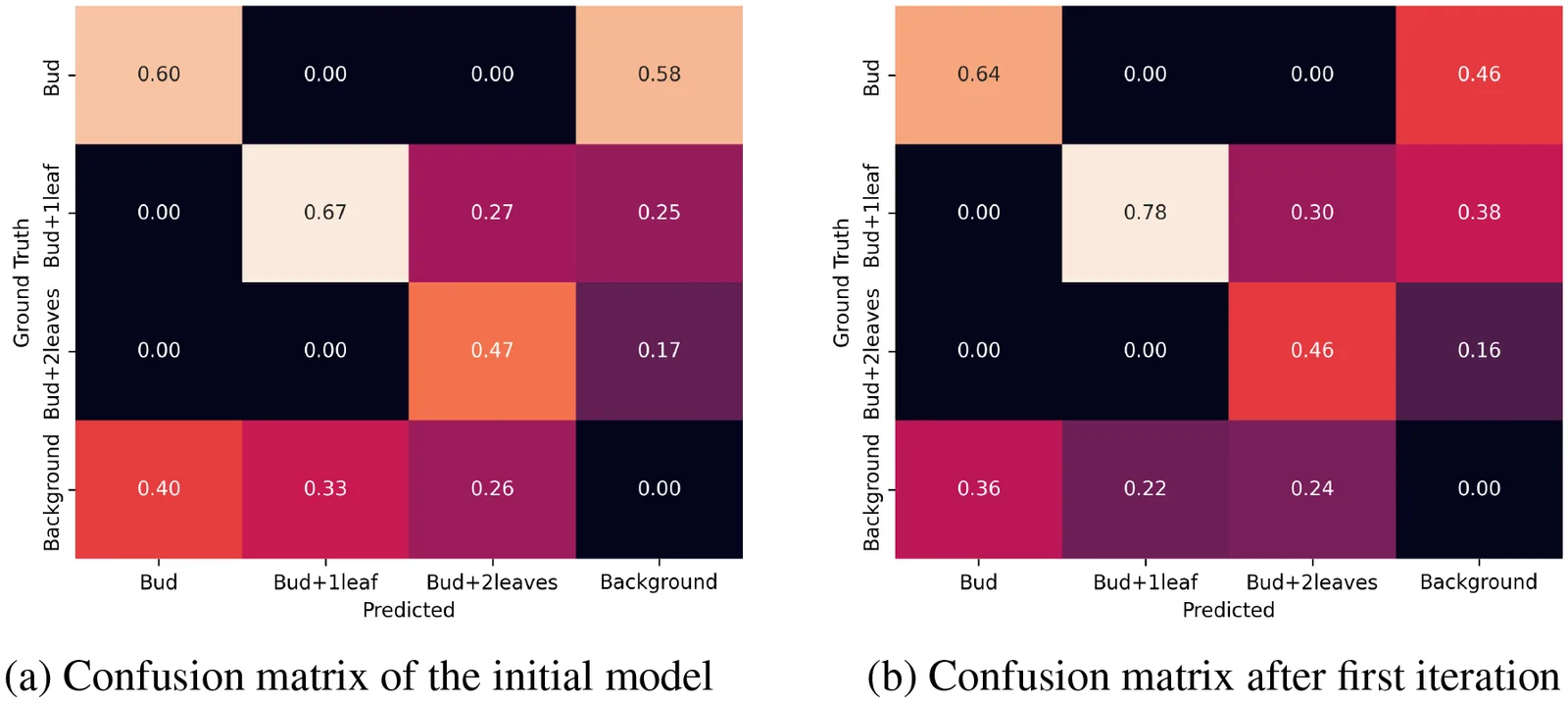
Confusion matrices produced by the initial model **(a)**, and the model after adding five images selected by the proposed active learning method **(b)**.

## 4 Discussion

The results suggest that the effectiveness of active learning is closely related to the ability of the uncertainty metric to identify samples that provide the greatest incremental information for model refinement. Instead of uniformly increasing the size of the training dataset, uncertainty-guided selection concentrates annotation effort on images where the model is less confident, allowing subsequent training iterations to correct decision boundaries more efficiently. Consequently, each annotation iteration contributes more to performance improvement than randomly selected samples, particularly during the early and intermediate stages of the active learning process.

Another important observation is the influence of imaging geometry on segmentation performance. The top-down (90°) viewpoint consistently provides more reliable predictions because tea buds and leaves are less affected by self-occlusion and exhibit more complete structural features. In contrast, the oblique (45°) viewpoint introduces greater variability in object appearance owing to overlapping foliage, partial visibility, and illumination changes. Under these more challenging conditions, uncertainty-guided sampling becomes increasingly valuable, as it preferentially selects difficult examples that are underrepresented in the current training set, leading to more rapid improvement than Random sampling.

The observed stagnation at approximately 0.70 mAP@0.5 in the oblique view suggests a performance ceiling, which we attribute primarily to data complexity rather than model capacity. The oblique view presents substantial challenges: heavy occlusion, perspective distortion, severe class imbalance, and small object sizes that would persist even with a larger backbone. This interpretation is corroborated by the top-down view achieving substantially higher accuracy (0.785 mAP) with the same architecture, confirming that viewpoint geometry and associated data complexity are the dominant factors defining the performance ceiling in this task.

The comparison between the two uncertainty metrics further highlights the trade-off between prediction quality and computational cost. Entropy considers the complete class probability distribution and therefore provides a richer description of predictive uncertainty. However, computing entropy requires additional processing of class probabilities for every detected instance. In contrast, the proposed mean confidence criterion can be directly derived from the detector output without additional probability calculations, resulting in a simpler and more computationally efficient sample selection process. Despite its structural simplicity, the proposed strategy achieves a similar segmentation accuracy to the Entropybased approach. This indicates that the additional computational complexity of full entropy estimation provides limited practical benefit for this application, making our simpler metric a more optimal choice.

These findings have practical implications for intelligent tea production research. Since only a small number of newly selected images are required during each active learning iteration, the proposed framework can be incorporated into an iterative annotation workflow in which field images are periodically collected, labeled, and used to update the segmentation model. Such a workflow enables model refinement without requiring complete dataset reannotation, offering potential benefits for resource-constrained annotation scenarios. More generally, the proposed framework provides an efficient strategy for reducing annotation costs while maintaining competitive segmentation performance and could be adapted to other agricultural vision tasks involving limited labeled data and evolving field conditions.

### 4.1 Limitations and future work

Contextualization with Prior Work: Our findings align with and extend several established results in the active learning literature, corroborating foundational paradigms Settles (2009) and domain-specific studies in plant phenotyping Rawat et al. (2022) where uncertainty-based sampling consistently outperforms random selection. Notably, the performance gap between our proposed method and random sampling is substantially wider under the challenging oblique (45°) view (final mAP@0.5: 0.697 vs. 0.550, a 26.7% relative gain) than under the conventional top-down view (0.785 vs. 0.670, a 17.2% relative gain). This disparity demonstrates that uncertainty-based active learning yields the highest utility precisely when the baseline model is weak or facing high visual complexity, a finding that echoes Gal and Ghahramani (2016) and provides empirical justification for prioritizing active learning workflows under difficult real-world imaging conditions. While our singlemetric mean confidence approach is computationally efficient, prior work indicates that such metrics can be susceptible to model miscalibration or varying class-conditional confidences Guo et al. (2017).

Uncertainty Metrics and Hybrid Sampling Strategy: Although the framework’s inherent sampling bias naturally up-weights underrepresented minority classes, the primary limitation of pure uncertainty-based methods (such as our proposed mean-confidence or entropy-based sampling) remains their reliance on instance-level prediction scores, which overlook the global feature distribution Settles (2009); Gal and Ghahramani (2016). Selecting the top*k* most uncertain samples without an explicit diversity constraint introduces structural vulnerabilities to batch redundancy, where multiple chosen images share identical failure modes like similar environmental occlusions. To resolve this bottleneck, our future work will pivot to a hybrid, two-stage sampling strategy directly motivated by successful multicriterion applications in biomedical imaging Zhao et al. (2025) and streaming learning Saran et al. (2023). For example, one possible approach is to combine k-means clustering with our proposed mean-confidence metric to jointly exploit diversity and uncertainty. The pipeline first selects the top-2*k* most uncertain samples based on the proposed mean-confidence metric (or an entropy-based metric). It then applies k-means clustering in the YOLO11 feature space to identify *k* diverse representative images, thereby ensuring that the selected samples are both highly informative and visually diverse.

Dataset size and generalization: The experimental dataset is relatively small (59–60 annotated images per viewpoint) and was collected at a single tea plantation (Fuliang County, Jingdezhen) on a single date (8 April 2025) under favorable natural daylight. While this scale is understandable given the high cost of fine-grained segmentation annotation (approximately 2 minutes per image), it limits the generalizability of the reported results to other tea varieties, growth stages, geographic regions, and seasonal conditions. To address this, we plan to: (1) expand the dataset through multi-site collection across at least three tea-producing regions with distinct cultivars and climate conditions; (2) include images captured across multiple growth stages (early spring, late spring, summer) to cover phenological variation; and (3) validate the framework on publicly available tea/plant segmentation benchmarks where available, or release our own dataset to facilitate community benchmarking (see Data Availability Statement below).

Failure Mode Analysis: To guide our future work, we plan to conduct a qualitative failure mode analysis on residual errors where the model underperforms (mAP@0.5 *<* 0.5) even after completing all active learning iterations. We anticipate identifying five primary environmental and morphological bottlenecks: (1) severe occlusion, where overlapping leaves or branches block tea buds, particularly in the oblique (45°) view; (2) extreme lighting conditions, where strong direct sunlight creates harsh shadows and overexposure, while low light reduces local foreground-background contrast; (3) wind-induced motion blur, which degrades edge sharpness and compromises the localization of flexible shoots; (4) atypical morphology, where tightly folded, early-stage immature buds or deformed shoots escape detection; and (5) dense clusters, which can cause individual adjacent masks to merge or generate conflicting overlapping predictions in far-range views. Our upcoming research agenda will address these anticipated limitations by exploring complementary strategies beyond active learning alone, specifically focusing on integrating robust data augmentations to simulate motion blur and photometric distortions, alongside multi-frame temporal fusion to disambiguate occluded structures across short video sequences. In addition, model capacity may influence the achievable segmentation performance, particularly for challenging samples with severe occlusion, dense clusters, and atypical morphology. Future work will therefore evaluate larger YOLO11 variants, such as YOLO11m-seg and YOLO11lseg, to determine whether increased model capacity can further improve segmentation accuracy and whether the annotation-efficiency benefits of the proposed active learning strategy remain consistent across different model scales.

Real-World Deployment Considerations: Translating the proposed framework from an experimental setup to operational tea-harvesting robots presents several practical challenges that form the core of our future engineering work. First, we plan to address variable illumination (e.g., dawn, dusk, and artificial night lighting) through domain adaptation or ondevice fine-tuning to prevent performance degradation outside controlled daylight conditions. Second, to handle wind-induced plant movement in hilly regions, we will explore lightweight temporal consistency modules or optical-flow-based motion compensation to stabilize real-time harvesting operating at 20–40 frames per second. Third, while the lightweight YOLO11n-seg model matches our strict real-time and hardware constraints, requiring under 10ms of inference latency and an ultra-small 6MB footprint suitable for embedded edge platforms like the NVIDIA Jetson Orin NX. Future work will systematically profile and optimize the entire end-to-end pipeline (including image acquisition, post-processing, and actuator control) for power consumption and throughput. Finally, we will adapt our active learning framework into a continual learning system to mitigate environmental and seasonal model drift. By queuing low-confidence deployment samples for periodic expert review, the system can autonomously adapt to shifting plant phenologies or pruning variations, lowering the total cost of ownership and accelerating the transition of vision-guided harvesters from research prototypes to field-deployable tools.

## Data Availability

The raw data supporting the conclusions of this article will be made available by the authors, without undue reservation.

## Footnotes

1
https://www.cvat.ai/

2
https://github.com/WenzhiLiao/active-learning-tea-segmentation

3
https://huggingface.co/Ultralytics/YOLO11/blob/97c61428146e92d06e5cde0c70308ed8d36a6c34/yolo11n-seg.pt
